# Characterization and expression of Rubisco activase genes in *Ipomoea batatas*

**DOI:** 10.1007/s11033-013-2744-7

**Published:** 2013-09-25

**Authors:** Yusong Jiang, Jianxi Wang, Xiang Tao, Yizheng Zhang

**Affiliations:** Key Laboratory of Resource Biology and Eco-environment of Ministry of Education, Sichuan Key Laboratory of Molecular Biology and Biotechnology, College of Life Science, Sichuan University, Chengdu, 610064 People’s Republic of China

**Keywords:** Gene expression, Protein interaction, Rubisco activase, Sweet potato, Two-dimensional electrophoresis

## Abstract

Two-dimensional electrophoresis, coupled with MALDI-TOF-MS, was used to identify differentially expressed proteins between young and mature leaves of sweet potato [*Ipomoea batatas* (L.) Lam]. The results showed that there were 25 differential proteins between young and mature leaves. The Rubisco activase (RCA) that catalyzes the activation of Rubisco in vivo and plays a crucial role in photosynthesis was among these 25 proteins. So far, little was known about the molecular biology of RCA in sweet potato. Here, this research reports the cloning and characterization of two genes encoding the short isoform and the long isoform of sweet potato RCAs. Analysis of DNA sequences of RCA suggested that the corresponding mRNAs were transcribed from two different genes. To study the roles of these two RCA isoforms in photosynthesis, we investigated the expression patterns of these RCA genes at the mRNA and protein levels every 2 h in a photoperiod and under different temperatures conditions. The results indicated that these two RCA isoforms may play different roles in regulating photosynthesis and they may be regulated by light, heat or both. In addition, there were interactions between Rubisco large subunit (RBCl) and short isoform RCA (RCAs) as well as RCAs and long isoform RCA (RCAl), but no interaction between RBCl and RCAl, implying they might form a sandwich-like structure (RBCl–RCAs–RCAl), at least in yeast cells. These results provided new information on the modulation of RCA genes in sweet potato, which could be useful in improving photosynthesis and plant growth in sweet potato.

**Electronic supplementary material:**

The online version of this article (doi:10.1007/s11033-013-2744-7) contains supplementary material, which is available to authorized users.

## Introduction

Sweet potato is the sixth most important crop for food and industrial materials worldwide after rice, wheat, potato, maize and barley, and it is the fifth most important crop in many developing countries (http://cipotato.org/sweetpotato/facts and http://www.fao.org/). Sweet potato is a cash crop with high yield, drought tolerance and wide adaptability to various climates and farming systems [1, 2]. Recently, sweet potato was used for ethanol production to lessen our dependence on fossil fuel [3, 4]. Sweet potato can be grown in areas that are not suitable for corn (a major crop for ethanol production in some countries) and yields 30 % more starch than corn [5]. Thus, more and more studies on sweet potato are focused on increasing biomass production by improving photosynthesis.

Leaf is the general location of photosynthesis and acts as a source of carbohydrate for sink nutrients to support growth in sink organs of plants. Leaf maturation is an important event in the process of leaf development and is closely related to photosynthesis efficiency, which is regulated by various proteins [6]. Rubisco (EC 4.1.1.39) is a key enzyme of photosynthetic carbon assimilation, and catalyzes the first step of photosynthetic carbon assimilation and photorespiratory pathways [7–9]. Rubisco exists in three states in photosynthetic cells: (1) inactive form (type E); (2) inactive form of carbamylation (type EC); (3) active form (type ECM). The proportion of these Rubisco forms directly influences photosynthesis in plants [10–12]. Rubisco activase (EC 4.1.1.36), a chloroplast protein encoded by nuclear genome, is one of the key enzymes that regulates the activity of the entire Calvin–Benson cycle via regulation of the activity of Rubisco [13]. It is generally assumed that RCA can alter the activity of Rubisco by facilitating the dissociation of the tightly bound sugar phosphates from Rubisco in a process that requires ATP hydrolysis, decreasing the threshold value of CO_2_ concentration for catalyzing the reaction carbamoyl and improving the activity of Rubisco significantly [14]. Previous study indicated that the initial carboxylation activity of Rubisco is positively correlated with the activity and content of RCA in mature leaves [15]. For transgenic plants expressing anti-RCA gene, the RCA content and Rubisco activity were all decreased, and meanwhile their photosynthesis was reduced dramatically. Moreover, after heat-stress at 42 °C, compared with the transgenic plants expressing anti-RCA gene, the photosynthesis of wild-type plants was recovered completely in a short time, while antisense plants only slightly recovered [16, 17].

In most plants, two isoforms of RCA (RCAs and RCAl, with molecular masses of 41–43 and 45–46 kD, respectively) are present, and they differ only at the C terminus. Unlike the RCAs, the RCAl holds a C-terminal extension that contains the redox-sensitive Cys residues [18, 19]. Two RCA isoforms can be produced by alternative splicing at the 3′ end of the pre-mRNA, giving rise to two transcripts with about 100 nt difference or may be produced from two separate genes [18, 20]. At present, it is not clear whether the two RCA isoforms have different physiological functions or whether they both play important roles in photosynthesis. Two spinach RCA isoforms arising from alternative splicing were capable of promoting Rubisco activation, but they showed remarkable differences in enzyme activities [21]. Vargas-Suárez et al. [22] reported that the accumulation of two maize RCA polypeptides, encoded by two separate genes, was regulated during leaf development. The activity of RCAl was regulated by redox state via thioredoxin f [23, 24], and this redox regulation was due to an interaction between carboxy extension and nucleotide-binding pocket in RCAl. Although rice leaves accumulate more RCAs than RCAl [25 ], it is unknown whether the two isoforms are changed during leaf development and what is the relationship between these RCA isoforms and photosynthetic capacity.

It is clear that, sweet potato exhibits the characteristics of efficient and longstanding photosynthesis even they are grown under stress conditions. Do these fine characteristics of sweet potato have relationships with these two isoform RCA? In order to better understand the mechanism of efficient photosynthesis in sweet potato, we studied expression patterns of Rubisco and RCA by differential proteomics analysis and reverse transcriptase polymerase chain reaction (RT-PCR) at every 2 h in a photoperiod as well as under the conditions of different light intensity and temperatures. Furthermore, we investigated the interactions of Rubisco large subunit with RCA short isoform and RCA long isoform.

## Materials and methods

### Plant materials

Sweet potato cv. *Xushu* 18 was used in this study. Plants were grown on test field under natural conditions in May of 2009 at Sichuan University. For 2-DE analysis, we harvested the unexpanded young leaves and the fully expanded mature leaves (Supplementary Fig. S1) at midday and frozen immediately in liquid nitrogen. For cloning of the two RCA isoform genes, the fully expanded leaves of sweet potato were collected. To investigate whether mRNA’s expressed pattern of *Ib*-*RCA* in leaf tissue is regulated by light, we harvested mature leaves every 2 h throughout a 24 h period. In order to investigate the temperature responses of *Ib*-*RCA* genes, sweet potato growing in field was transplanted to climate box (25 °C under a 14/10 h light/dark photoperiod and 200 μEm^−2^ s^−1^). After continuous light (25 °C, 200 μEm^−2^ s^−1^) treatment for 2 days, sweet potato was then treated at different temperatures (20, 25, 30, 35 °C and 200 μEm^−2^ s^−1^) for 24 h. We sampled and frozen immediately in liquid nitrogen, then stored at −80 °C until use. Sequences data from this article have been deposited at GenBank (http://www.ncbi.nlm.nih.gov) under accession numbers JQ923423—JQ9234231.

### Extraction of total proteins

Proteins were extracted from 1 g of leaf tissues using a two-step precipitation/extraction method [26]^.^ Leaf tissues were ground in a mortar in liquid nitrogen and then suspended in acetone containing 10 % trichloroacetic acid and 0.07 % β-mercaptoethanol. Proteins were precipitated overnight at −20 °C. The precipitate was washed twice or more with 0.07 % β-mercaptoethanol in acetone and then dried. The dried pellet was resuspended in 100 mM Tris–HCl buffer (pH 8.0), 10 mM EDTA, 30 % sucrose, 2 % CHAPS, 2 % SDS, 10 mM PMSF and 2 % β-mercaptoethanol, and extracted with equal-volume of Tris-saturated phenol. Proteins were precipitated overnight at −20 °C by adding 4 volumes of 0.1 M ammonium acetate in methanol. The pellet was washed at least twice with ammonium acetate/methanol and 0.07 % β-mercaptoethanol in acetone, and dissolved in 2-DE sample buffer containing 7 M urea, 2 M thiourea, 2 % CHAPS (w/v), 1 % dithiothreitol (w/v), 0.5 % 3/10 ampholyte (v/v), 0.2 % 7/11 ampholyte (GE Healthcare, California, USA), 0.002 % bromphenol blue. Total protein was analyzed using the methods of Bradford [27].

### 2-DE and data analysis

Isoelectric focusing electrophoresis (IEF) was carried out by using an IPGphor II electrophoresis system (GE Healthcare) and 24-cm immobiline dry strips, with nonlinearity pH gradient of 3–10 (GE Healthcare). Protein samples (300 μg) were loaded. IEF was performed by step at 30 V for 12 h, gradient to 200 V for 2 h, and gradient to 500 V for 1 h, gradient to 8,000 V for 1 h, and step at 8,000 V, until a total of 48 kVh were reached. Prior to the SDS-PAGE, the gel strips were equilibrated twice as described [28]. After equilibration, the strips were transferred to vertical SDS-PAGE system (12.5 % resolving gel), perform electrophoresis at 20 °C, first for 45 min at 1 W per strip and then 15 W per strip. After electrophoresis, gels were stained with coomassie blue (CBB) staining. The intensities of the increased or decreased protein spots from samples were scanned as digitalized images by using a UMAX scanner (UTA-1100; GE Healthcare). The optical resolution is 300 dpi. Spots were detected and quantified with the Imagermaster software (Version 5.0; GE Healthcare), on the basis of their relative volume (the spot volume was divided by the total volume over the whole set of gel spots). After spots were detected and quantified, matching and editing were carried out. All experiments were repeated two times.

### In-gel digestion, MS and database searching

Proteins were excised from the CBB-stained gel, sliced into pieces and washed with 50 mM ammonium bicarbonate in 50 % ACN for destaining. The proteins were then digested in-gel with trypsin (Roche, Italy), as described by Hellmann et al. [29]. The digested peptide solution (3 μL) was taken for MALDI-TOF-MS analysis (Bruker, USA). Protein identification was performed by searching the National Center for Biotechnology Information nonredundant Greenplant database (NCBInr) using MASCOT program (http://www.matrixscience.com) and our local transcriptomic database of sweet potato by GPMAW 8.0 software (http://www.gpmaw.com). The following parameters were used for database searches: peptide tolerance, 50 ppm; MS/MS tolerance, 0.5 Da; maximum allowed missed cleavage, 1; fixed modification, carbamidomethylation of cysteine; variable modification, methionine oxidation. Protein scores were derived from ions scores as a nonprobabilistic basis for ranking protein hits and the protein scores as the sum of a series of peptide scores. The score threshold to achieve *p* < 0.05 is set by program algorithm, and is based on the size of the database used in the search.

### Isolation of RNA and genomic DNA

Total RNAs were isolated from leaves using Trizol reagent (Invitrogen, California, USA) according to the instruction manual and was treated with RNase-free DNase I (TaKaRa, Dalian, China). RNA concentrations were determined using a spectrophotometer (260/280). After heating at 65 °C for 5 min to denature RNA and inactivate RNases, 1 μg of total RNAs were subjected to reverse transcription using 200 units of M-MLV reverse transcriptase (TaKaRa), 40 units of RNase inhibitor (TaKaRa), 100 ng of oligo (dT)_12–18_ primer, 10 mM (each) dNTPs, 4 μL reverse transcriptase buffer, in a total volume of 20 μL at 42 °C for 1 h. The reaction was terminated by heating at 70 °C for 15 min. Genomic DNA was extracted from mature leaves of sweet potato using the cetyl-trimethyl-ammonium bromide protocol as described by Weising et al. [30].

### Cloning of *Ib*-*RCA*

According to the results of MALDI-TOF-MS and the local transcriptomic database of sweet potato [31], primers were designed (Supplementary Table S1) to amplify the cDNA and genomic DNA fragments of *Ib*-*RCAs* (for the sequence of the short *RCA* ORF) and *Ib*-*RCAl* (for the sequence of the long *RCA* ORF) using KOD FX DNA polymerase (TOYOBO, Shanghai, China).

### Semiquantitative and quantitative PCR

For semi-quantitative polymerase chain reaction (PCR), half a microliter of the first strand cDNA products were used for each gene amplification. PCR conditions were as follows: denaturation at 94 °C for 30 s, annealing at 60 °C for 30 s, and an extension step at 72 °C for 1 min. Linearity between the amount of input RNA and the final PCR products was verified. The transcriptional level of *β*-*actin* was used as an internal control. Specific primers (Supplementary Table S1) were designed according to gene sequences of MALDI-TOF-MS-identified proteins and the local transcriptomic database of sweet potato. The real-time PCR was performed by the IQ5 real-time PCR system (BIO-RAD, California, USA) in a total volume of 20 μL containing 100 ng of cDNA template, 1× SYBR^®^ *Premix Ex Taq*™ II (Perfect Real Time, TaKaRa), and a 400 nM concentration of each primer. Serial dilutions of each cDNA were used to generate a quantitative PCR standard curve to calculate the corresponding PCR efficiencies. After initial denaturation at 95 °C for 30 s, the amplification was carried out through 40 cycles, each consisting of denaturation at 95 °C for 5 s, primer annealing at 60 °C for 30 s, and DNA extension at 72 °C for 30 s. Melting curves were obtained, and quantitative analysis of the data was performed using the BIO-RAD IQ5 standard edition optical System software (version 2.1) in a normalized expression (ddCt) model. Transcriptional levels of *Ib*-*RCAs* and *Ib*-*RCAl* were normalized to *β*-*actin* gene. Specific primers of *Ib*-*RCAs*, *Ib*-*RCAl* and *β*-*actin* for real-time PCR were list in Supplementary Table S1. Results were obtained from three biological replicates.

### Protein gel blot analysis

Protein extracts were subject to SDS-PAGE analysis using 12 % acrylamide resolving gel (Mini Protean II System; Bio-Rad). Separated proteins were then transferred to polyvinylidene difluoride membranes, and nonspecific binding of antibodies was blocked with 5 % nonfat milk in phosphate-buffered saline solution (pH 7.4) for 2 h at room temperature. Membranes were then incubated overnight at 4 °C with polyclonal anti-RCAc (or anti-RCAe) antibodies. Anti-RCAc antibodies (recognize the common sequence of two Ib-RCA isoforms) and Anti-RCAe antibodies (recognize the specific carboxyl terminal sequence of Ib-RCAl) were generated by immunizing male New *Zea*-*land* rabbits with the purified partial peptides of Ib-RCA. The DNA sequences used for Ib-RCA peptides in preparation of antibodies were list in Supplementary Table S2. The crude antisera of anti-RCA and anti-NAPDH (HuaAn; Hangzhou, China) were used at dilutions of 1:200 and 1:4,000. Anti-rabbit IgG antibodies coupled to horseradish peroxidase were used as the secondary antibodies. The enhanced chemiluminescence Western-blotting detection kit (TianGen, Beijing, China) was used for color development. Immunoblots were scanned and densitometric analysis was performed using the Bio-Rad Quantity One software (version 4.6.2). Each reaction was performed in triplicate.

### Protein–protein interactions assays

Yeast two-hybrid assays were carried out using a GAL4-based yeast two-hybrid system (MATCHMAKER Two-Hybrid System 3; Clontech, USA). The truncated cDNA sequences contained an initiator codon and a completely coded mature peptide sequence of *Ib*-*RBCl*, *Ib*-*RCAs* and *Ib*-*RCAl,* and were amplified by PCR. The truncated *Ib*-*RBCl* (or *Ib*-*RCAs*) was then digested with two restriction enzymes *Nco* I and *Bam*H I, and inserted into the pGBKT7 vector (Clontech) to generate a construct of *Ib*-*RBCl* (or *Ib*-*RCAs*) cDNA fused in-frame to the GAL4 DNA-binding domain (amino acids [a.a.] 1–147 of GAL4) as the bait. The truncated *Ib*-*RCAs* (or *Ib*-*RCAl*) was digested with two restriction enzymes *Nco* I and *Eco*RI, and inserted into the pGADT7 vector (Clontech) to generate a construct of *Ib*-*RCAs* (or *Ib*-*RCAl*) cDNA fused in-frame to the GAL4 activation domain (AD) (amino acids [a.a.] 768–881 of GAL4) as the prey. Both the bait and the corresponding prey plasmids were used to co-transform AH109 yeast strain and selected on dropout medium lacking leucine and tryptophan (SD/-Leu-Trp) because the pGBKT7 and pGADT7 vector had selectable TRP1 and LEU2 marker. Positive clones were further tested by colony-lift filter assay with X-β-gal and selected on Quadruple Drop Out medium (SD/-Leu/-Trp/-His/-Ade, high stringency selection) containing 20 mM 3-aminotriazole (3-AT), a competitive inhibitor of the His3 protein to increase the stringency of selection. Only the blue clones by colony-lift filter assay and growth normally on Quadruple Drop Out medium were picked for further tests. Co-transformants of pGBKT7-murine p53 and pGADT7-SV40 large T antigen were used as a positive control, and yeast cells with various empty vectors (no insert) were used as a negative control. The primers using in Yeast two-hybrid assays were list in Supplementary Table S1.

## Results

### Differential expression of sweet potato proteins in young and mature leaves

**Fig. 1:**
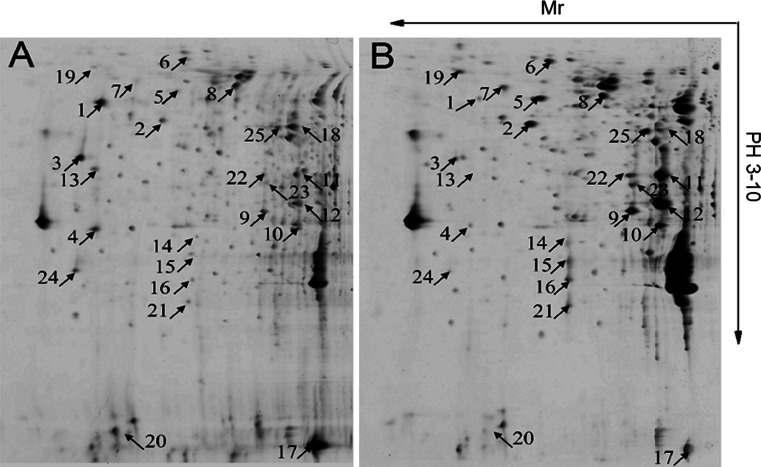
Protein expression patterns in young leaves (**a**) and mature leaves (**b**) of sweet potato. Proteins were extracted from the young and mature leaves, separated by 2DE gels (24 cm IPG strip, pH3-10, 12.5 % SDS-PAGE), and stained by CBB-staining. The *numbers* indicate the differentially expressed proteins between young and mature leaves. The relative levels of protein expression were analyzed by imagemaster 5.0 software

Proteomic characterization of sweet potato leaves was achieved by 2-DE analysis of proteins extracted from leaves at young and mature stages. Representative gels were shown in Fig. 1. There was 806 and 753 protein spots detected in young and mature leaves, respectively. Relative intensity analysis of protein spots by Imagemaster 5.0 software displayed that 565 protein points were matched and the related coefficient was 0.798 (data not shown). To evaluate the differentially expressed proteins, we performed statistical analysis with these data using the student *T* test and found 25 differentially expressed proteins with good reproducibility (the differences were more than 2.5-fold; *p* < 0.05). Seventeen out of 25 proteins were up-regulated, and the other 8 were down-regulated (Fig. 1). All 25 proteins were excised from gels, digested and subjected to MALDI-TOF–TOF/MS analysis. Since there was no available genome sequence of sweet potato, identification of sweet potato spots by MALDI-TOF–TOF-MS experiments proved challenging. Therefore, homology searches were carried out in NCBI GreenPlants database and the transcriptomic database of sweet potato [31]. The functions of 20 proteins were known, but the other five proteins could not be identified. Among the 20 known proteins, 13 were up-regulated and 7 were down-regulated (Table 1). Based on their functions, these 20 proteins were classified into six categories: photosynthesis, energy metabolism, transcription regulation, stress-responsive, skeleton protein, and transport protein (Supplementary Table S3).

**Table 1:** Differentially expressed proteins identified by MALDI-TOF–TOF-MS

Spot no.^a^	Accession no.	Homologous protein	Species	Score^b^	Coverage(%)^c^	Theoretical Mr/PI	Change (fold)	Amino acid sequenced^d^
1	gi|255585710	Zinc/iron transporter	*Ricinus communis*	74	69	18,797/8.29	−17.6	F.IGLDVQIVVLGTG.K
2	gi|1708311	Stromal 70 kD heat shock-related protein	*Spinacia oleracea*	196	7	64,918/4.87	5.8	K.LSFSDLDEVILVGGSTR.I
3	gi|6911146	Putative glycine-rich RNA-binding protein 2	*Catharanthus roseus*	94	36	16,312/7.82	−8.1	R.REGGGGYGGGG.R
5	gi|8131593	oxygen evolving enhancer protein 2	*Bruguiera gymnorrhiza*	80	12	17,583/4.91	7.4	E.VEFPGQVL.R
7	gi|195622374	Fructose-bisphosphate aldolase	*Zea mays*	197	30	40,506/5.39	6.3	R.LDSIGLENTEAN.R
9	gi|67079068	Rubisco large subunit	*I. lacunosa*	125	42	25,725/8.55	7.6	D.TDILAAF.R
10	gi|3328122	Phosphoglycerate kinase precursor	*Solanum tuberosum*	184	31	50,594/7.68	3.2	L.ASLADLFVNDAFGTAH.R
11	gi|162312077	Rubisco activase	*I. batatas*	427	48	48,637/8.16	8.4	F.VAGLGVEKVNE.R
12	gi|162312077	Rubisco activase	*I. batatas*	336	32	48,637/8.16	12.3	S.FQCELVF.R
13	gi|2498076	Nucleoside diphosphate kinase	*Helianthus annuus*	86	12	16,206/6.3	−9.5	G.DFAIDIG.R
16	gi|2443881	Contains beta-transducin motif	*A. thaliana*	72	18	130,788/8.84	3.5	K.LLHEFKFHEGPIR.S
17	gi|5917747	Elongation factor-1 alpha subunit	*Lilium longiflorum*	238	32	49,653/9.15	−12.9	E.HALLAFTLGV.R
18	gi|1244566	Acetyl-CoA carboxylase	*Triticum aestivum*	77	14	252,831/6.01	−3.7	R.KIPLIYLSATAGAR.L
19	gi|56407431	Granule bound starch synthase	*Lycianthes amatitlanensis*	75	36	15,939/5.35	4.2	F.IGLDVQIVVLGTG.K
20	gi|145049729	Cyclophilin	*I. batatas*	203	62	18,579/7.66	−6.4	V.FFDMTIGGQPAG.R
21	gi|47606728	Carbonic anhydrase	*Flaveria bidentis*	123	23	35,922/5.85	2.7	E.AVNVSLGNLLTYPFV.R
22	gi|85679505	Phytochrome B 2	*P. trichocarpa*	81	1.84	127,849/6.04	3.0	L.VGEIFGSC CRLKGPDA.M
23	gi|15218281	F-type H^+^-transporting ATPase subunit gamma	*A. thaliana*	135	13	42,937/6.7	2.8	A.LQESLASELAS.R
24	gi|224088196	Actin 3	*P. trichocarpa*	440	59	41,902/5.31	−3.2	K.SYELPDGQVITIGAER.F
25	gi|170125	Phosphoribulokinase	*Spinacia oleracea*	202	25	45,839/5.97	2.6	I.LVIEGLHPMYDA.R

^a^The spot numbers correspond to protein spot numbers as given in Fig. 1

^b^The score of the result of [− 10 × log (*p*)] is lower than the significance threshold level (*p* < 0.05)

^c^Percentage of sequence coverage of identified peptides for proteins in NCBI GreenPlants database

^d^The sequence of matched peptides by MALDI-TOF–TOF-MS

### Transcript levels in young and mature leaves

**Fig. 2:**
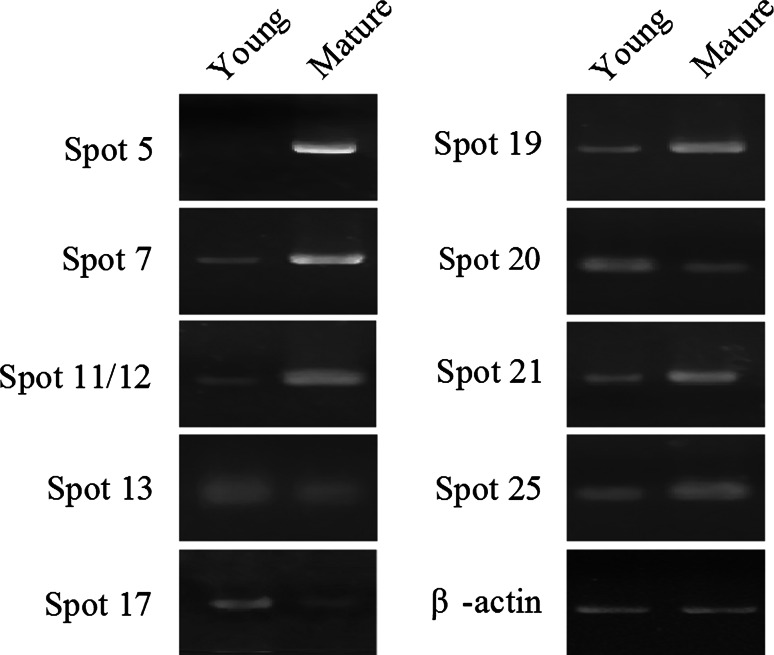
Semi-quantitative RT-PCR analysis of genes differentially expressed in young and mature leaves. The products of RT-PCR were separated on 1.2 % agarose gel. Spot 5, oxygen evolving enhancer protein 2; Spot 7, fructose-bisphosphate aldolase; Spot 11/12, Rubisco activase; Spot 13, nucleoside diphosphate kinase; Spot 17, elongation factor-1 α subunit; Spot 19, granule bound starch synthase; Spot 20, cyclophilin; Spot 21, carbonic anhydrase; Spot 25, phosphoribulokinase. The β-actin gene was used as an internal control

To investigate whether the proteins were correlated with their transcript levels, nine proteins with significant differences were chosen for further study by semi-quantitative RT-PCR. As shown in Fig. 2, the proteins and mRNAs appear to be generally correlated. However, there also were some variations at the protein and mRNA levels. For example, the expression of spot 13 (nucleoside diphosphate kinase), spot 20 (cyclophilin), spot 25 (phosphoribulokinase) in young leaves were about −9.5, −6.4, 2.6 times higher than those of the mature leaves at protein level, while at the mRNA level, the differences between the young and mature leaves were not striking (Figs. 1, 2).

### Cloning and sequence analysis of *Ib*-*RCAs* and *Ib*-*RCAl* cDNAs and genes

**Fig. 3:**
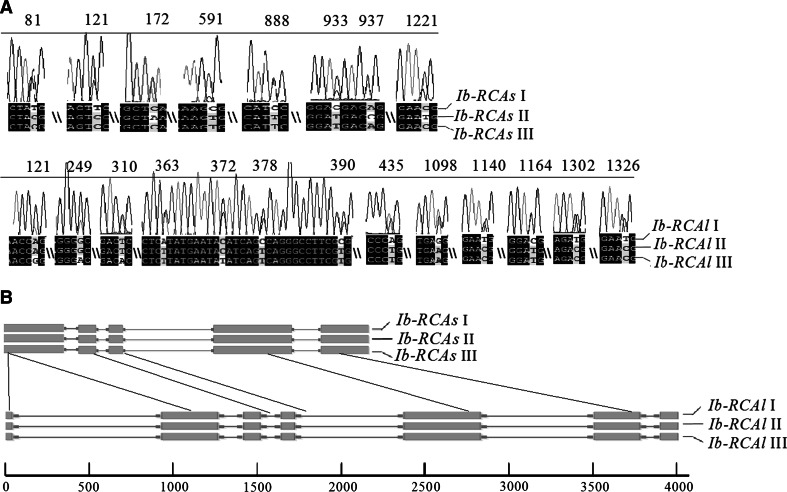
Structural features of the two *Ib*-*RCA* isoforms. **a** Nucleotide differences of the three *Ib*-*RCAs* homologous genes (named *Ib*-*RCAs* I, II, III) and the three *Ib*-*RCAl* homologous genes (named *Ib*-*RCAl* I, II, III). **b** Genomic structures of *Ib*-*RCAs* and *Ib*-*RCAl* genes. Exons (shown in *gray boxes*) and introns (shown in *white lines*) are drawn to scale

In order to understand the relationship between the efficient photosynthesis and Rubisco activase in sweet potato, two isoforms of *RCA* genes were cloned and sequenced (Fig. 3). The sequencing results indicated that the two isoforms contained a 1,320 bp ORF (named *Ib*-*RCAs*) corresponding to a deduced protein of 440 amino acid residues and a 1,455 bp ORF (named *Ib*-*RCAl*) of a deduced protein of 485 amino acid residues, respectively. It was also found that, there were probably three highly homologous isoforms (homology >99 %) in both *Ib*-*RCAs* (named *Ib*-*RCAs* I, II, III) and *Ib*-*RCAl* (named *Ib*-*RCAl* I, II, III) (Fig. 3a). To ensure that these changes were not due to experimental errors, direct sequencing of PCR products of the full-length ORFs also confirmed the existence of three highly homologous isoforms with two peaks at the same base location (Fig. 3a). Although nucleotide sequences of three similar isoforms had different numbers of SNP, most of them occurred in the first or third base of codons, so amino acid sequences changed rarely (Supplementary Table S4).

Genomic sequence of *Ib*-*RCAs* and *Ib*-*RCAl* were obtained by PCR using sweet potato genomic DNA as template. The fragments were cloned into pMD19-T and then sequenced. The full-length genomic sequences of *Ib*-*RCAs* and *Ib*-*RCAl* were 2217 and 4086 bp, including four and six introns, respectively (Fig. 3b). There also exist three homologous genome sequences with different numbers of SNP in both *Ib*-*RCAs* and *Ib*-*RCAl*; yet, the length and position of their introns are not different (Fig. 3b). The results of bioinformatics analysis indicated that although the distribution and position of introns were conserved in the two isoforms, sequences of the introns shared no homologies.

### Expression patterns of the two Ib-RCA isoforms

**Fig. 4:**
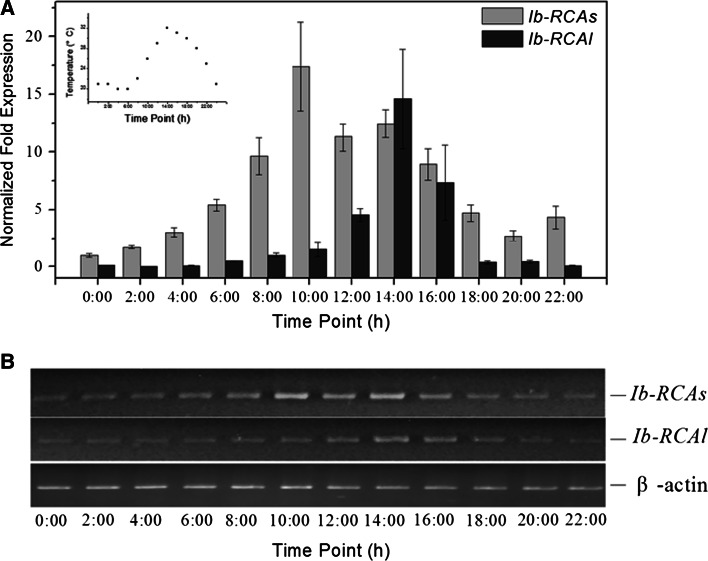
The expression patterns of *Ib*-*RCA* genes in a photoperiod. **a** Expression patterns of the *Ib*-*RCA* genes by relative quantitative real-time PCR as shown in *graphs*. RNA samples were collected at 2 h interval from 0 to 22 h. **b** Expression patterns of the *Ib*-*RCA* genes by semi-quantitative PCR as shown in gels. The β-actin was used as a control. Inside *graph* is the change of temperature in a photoperiod. Data (mean ± SE) are typical results from three independent experiments performed

**Fig. 5:**
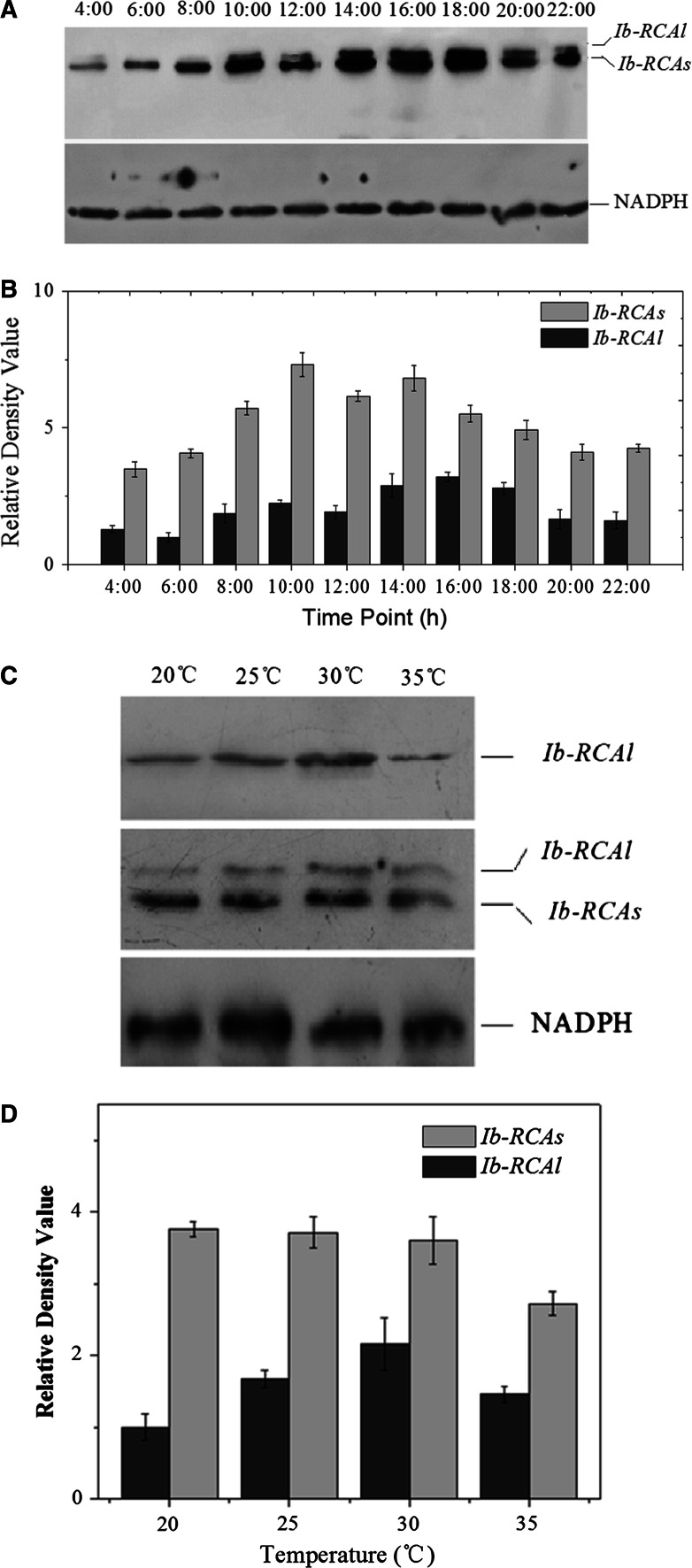
Protein gel blot analysis of Ib-RCA proteins in sweet potato. **a** The expression patterns of Ib-RCA in a photoperiod. **b** Relative densitometric analysis of Western blot gels using the Bio-Rad Quantity One software. **c** Expression patterns of Ib-RCA proteins under different temperatures. **d** Relative densitometric analysis of the Western blot gels. The polyclonal anti-RCAc antibodies can recognize two Ib-RCA isoforms concurrently, and the polyclonal anti-RCAe antibodies can only recognize Ib-RCAl. NAPDH was used as a control. Crude antisera of anti-RCAc, anti-RCAe and anti-NAPDH were used at dilutions of 1:200, 1:200 and 1:4,000, respectively. Data (mean ± SE) are typical results from three independent experiments performed. Statistical analysis was carried out using Origin software (version 8.0)

To study whether mRNA’s accumulation of *Ib*-*RCA* in leaf tissue is regulated by light, we determined the mRNA and protein levels of *Ib*-*RCAs/Ib*-*RCAl* by q-PCR and Western-blot. Before quantitative analysis, serial dilutions for each cDNA (10^−1^–10^−5^) were used to make a quantitative PCR standard curve to calculate the PCR efficiencies of *Ib*-*RCAs*, *Ib*-*RCAl* and *β*-*actin*. The standard curves demonstrated that the amplification efficiencies for *Ib*-*RCAs*, *Ib*-*RCAl*, *β*-*actin* of the real-time PCR were 98.2, 100.8, 98.8 %, respectively, and the values of R^2^ (correlation coefficient′s square) were 0.994, 0.966, 0.991, respectively (Supplementary Fig. S2). The relative quantitative PCR results of *Ib*-*RCAs* and *Ib*-*RCAl* showed that the maximal mRNA level of *Ib*-*RCAs* was detected at 10:00, 4 h after the beginning of the light period. The expression level decreased moderately at 12:00 compared with that at 10:00. However, the mRNA level of *Ib*-*RCAs* recovered slightly after 12:00, and then decreased gradually to the minimum level at midnight (Fig. 4). Compared with *Ib*-*RCAs,* the expression level of *Ib*-*RCAl* began to rise at 12:00 and reached the peak at 14:00, then decreased quickly (Fig. 4). Protein levels of these two Ib-RCA isoforms in the leaves of sweet potato shared similar characteristics at the mRNA levels. From 10:00, the Ib-RCAs accumulated quickly, declined gradually after 16:00, and then maintained at a comparatively low level at night. After 14:00, the accumulation of Ib-RCAl began to increase, and then declined after 16:00 (Fig. 5a, b).

In order to investigate the temperature responses of *Ib*-*RCAs*/*Ib*-*RCAl* genes, we sampled the leaves with different treatment and carried out protein gel blot analysis. Results demonstrated that the *Ib*-*RCAl* expression increased gradually from 20 to 30 °C, while declined dramatically at 35 °C (Fig. 5). It was also found that, at 20, 25 and 30 °C, the Ib-RCAs protein accumulation had no significant difference. However, when the temperature rose to 35 °C, Ib-RCAs’ expression declined significantly.

### Interactions of Ib-RBCl with Ib-RCAs or Ib-RCAl

**Fig. 6:**
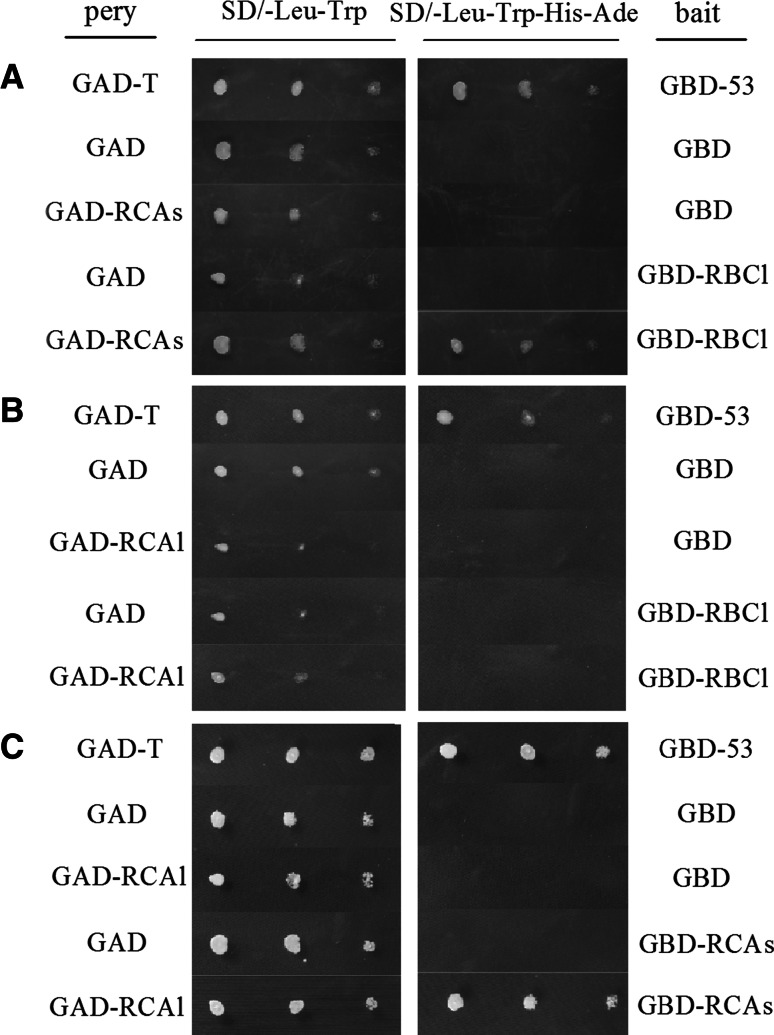
Yeast two-hybrid assay for detecting protein–protein interactions among Rubisco large subunit and two RCA isoforms. **a** The interaction of Rubisco large subunit with *Ib*-*RCAs.* **b** The interaction of Rubisco large subunit with *Ib*-*RCAl*. **c** The interaction of *Ib*-*RCAl* with *Ib*-*RCAs.* Yeast cells transformed with plasmids expressing murine p53 and SV40 large T-antigen were used as positive controls. Yeast cells transformed with various empty vectors (no insert) were used negative controls

To investigate whether both Ib-RCAs and Ib-RCAl could activate Rubisco, we studied the interactions of Ib-RBCl with these two isoform Ib-RCA. The yeast strain AH109 was co-transformed with bait plasmid pGBKT7-RCAs and prey plasmid pGADT7-RCAl, or co-transformed with bait plasmid pGBKT7-RBCl and prey plasmid pGADT7-RCAs, both formed blue colonies on filter paper by colony-lift filter assay with X-β-gal (data not shown) and the corresponding colonies grew normally on Quadruple Drop Out medium (Fig. 6). However, yeast AH109 strains co-transformed with bait plasmid pGBKT7-RBCl and prey plasmid pGADT7-RCAl could only grow on the plate of SD/-leu-Trp, and no blue yeast colonies appeared on filter paper by colony-lift filter assay (data not shown). Yeast cells co-transformed with various empty vectors (no insert) lacked any visible growth on Quadruple Drop Out medium (SD/-Leu/-Trp/-His/-Ade, high stringency selection), providing clear evidence that expression of Ib-RBCl, Ib-RCAs or Ib-RCAl alone did not lead to expression of the reporter genes in yeast. These results indicated that at least in yeast cell, there was interaction between Ib-RBCl and Ib-RCAs as well as Ib-RCAs and Ib-RCAl, and no interaction between Ib-RBCl and Ib-RCAl.

## Discussion

### Differential proteins expression in sweet potato leaves

Leaf maturation, as an important event in leaf development, is closely related to photosynthesis efficiency and regulated by various proteins. Data from 2D gel analysis indicated that some proteins were up-regulated and others were down-regulated in young and mature leaves. Due to lack of the complete genomic information of sweet potato, it was difficult to identify differentially expressed proteins by MALDI-TOF–TOF-MS. In order to obtain this information, the NCBI GreenPlants database and the transcriptomic database of sweet potato were used for homology analysis. Meanwhile, the data from semi-quantitative RT-PCR demonstrated that at both transcript and protein levels, these proteins had the same tendency of up- or down-regulation although the fold of increase or decrease was different (Figs. 1, 2). Because it is a complex process from mRNA to protein, both post-transcriptional and post-translational events could have caused the differences between mRNA and protein levels [32, 33]. Among the identified proteins, nearly 50 % were associated with photosynthesis in the process of leaf maturation, and most were up-regulated. This result suggests that the improvement of photosynthetic system was a key event in the process of leaf maturation, and the leaf goes through a transition from heterotrophic nutrition to autotrophic nutrition.

Rubisco encoded by both nuclear and chloroplast genome, is responsible for the photosynthesis and is the most important enzyme directly involved in the carbon fixation. Rubisco large subunit is the catalytic unit and can combine zymolyte (CO_2_ and RUBP) and Mg^2+^. However, the photosynthetic rate is not dependent on the content of the internal Rubisco but the activated state of Rubisco [34, 35]. At present, it is suggested that the main function of RCA is to promote the transition from the inactive Rubisco to active Rubisco [36]. Martinez et al. [37] found that Rubisco had high catalytic activity if it contained high level of RCA proteins in high yield maize cultivars. The contents of Rubisco and RCA appeared to be up-regulated during leaf maturation in sweet potato, which further supports their roles in photosynthesis.

In this study, we also found that some proteins playing roles in aspects of respiration, transcriptional regulation and stress response are also differentially expressed (Table 1), such as fructose-bisphosphate aldolase, nucleoside diphosphate kinase elongation factor-1 alpha subunit, putative glycine-rich RNA-binding protein 2, 70 kD heat shock-related protein etc. The physiological functions of changes in these proteins are not clear at this time.

### Three *Ib*-*RCAs* and *Ib*-*RCAl* genes in sweet potato

The mRNAs for sweet potato RCA s- and l-isoforms are transcripts of separate genes. In plant species studied so far, RCA proteins are present either as s-isoform and l-isoform or only s-isoform [13]. In plants such as Arabidopsis, spinach [20], and rice [25], there are two RCA isoforms from two mRNAs that are produced from alternative splicing of the transcribed pre-mRNA of a single RCA gene. However, cotton, maize, soybean are known to have two RCA isoforms that are encoded by different genes [18, 22, 38]. In this study, we also found that the mRNAs for sweet potato RCA s- and l-isoforms were transcribed from two different *RCA* genes (Fig. 3). This conclusion was supported by the following evidences: (1) *Ib*-*RCAs* contains four introns, while *Ib*-*RCAl* contains six introns. Although the distribution and position of these introns are conserved in *Ib*-*RCAs* and *Ib*-*RCAl*, the length and sequence of these introns are quite different; (2) the difference in amino acid composition between *Ib*-*RCAs* and *Ib*-*RCAl* could not be resolved by the current *RCA* transcript splicing mechanism observed in Arabidopsis, spinach, and rice; (3) the amino acid sequences of Ib-RCAs and Ib-RCAl showed approximately 75 % identity.

More recent studies revealed that there were three highly homologous genes for both *Ib*-*RCAs* and *Ib*-*RCAl*. Similarly, it was found that several homologous genes for sucrose transporter genes and 14-3-3 protein genes were present in sweet potato, which were likely from two or three subgenomes of hexaploid sweet potato [ 39–41 ]. The high homologies of these genes, which should be from three similar subgenomes, imply that the sweet potato is probably an autohexaploid origin. However, this inference cannot be confirmed at this time, because the wild ancestors of sweet potato have not been determined.

### Two Ib-RCA isoforms may have different roles in sweet potato

Unlike the two RCAs in Arabidopsis and spinach, which are expressed at about the same level [38], sweet potato leaf contains a much greater amount of s-isoform than l-isoform at the mRNA and protein levels (Figs. 4, 5), suggesting that the unequal amounts of the two RCA isoform in sweet potato leaf result from the differences of gene expression. Different protein abundance of two RCA isoforms might reflect different physiological functions. Expression of Ib-RCAs was induced by light (Fig. 4). Interestingly, expression of Ib-RCAs was repressed a little at noon time (Fig. 4a). As a result, the expression pattern was a two-peak curve in a day [2]. When light was controlled, the accumulation of Ib-RCAl was low at 20 °C, and was up-regulated significantly at a higher temperature. In contrast, the Ib-RCAs expression did not change obviously from 20 to 30 °C, whereas *Ib*-*RCA*s and *Ib*-*RCA*l expression both decreased significantly when the temperature reached 35 °C (Figs. 4, 5). Similar results were also found by Crafts-Brandner et al. [12] and Carmo-Silva et al. [42], who believed that the decline of the Rubisco activity might be caused by the inactivation rate of Rubisco exceeding reactivation capacity of Rubisco by RCA or the capacity of the combination of RCA and Rubisco decreasing, when the temperature reached at 35–40 °C . These evidences implied that *Ib*-*RCAs* may be regulated by light and the *Ib*-*RCAl* may be induced by heat. The result from protein–protein interactions assay indicated that there was interaction between *Ib*-*RCAs* and Rubisco large subunit (Fig. 6), which provided a further proof for Portis’s model of activation mechanism between RCAs and Rubisco large subunit [13]. Up to now, research of RCAl is scarce, yet it was reported that over-expression of RCAl could increase heat tolerance in rice and populus [21, 43]. Expression pattern of Ib-RCAs is different from that of Ib-RCAl in the process of photosynthesis and they may act as the complementary roles. Wang et al. [10] established a sandwich ELISA method for quantitative analysis of the interactive complex of RCA and RBCl based on specific monoclonal antibodies (mAbs) in rice, however, the knowledge of the RCAs/RCAl and RCAl/RBCl relationships was still very limited. Protein–protein interactions assay showed that Ib-RCAl has strong interaction with Ib-RCAs but Rubisco (Fig. 6). Furthermore, according to the thermal stability of Rubisco and RCA under heat stress [44–46] and the expression pattern of Ib-RCAs and Ib-RCAl during a photoperoid (Figs. 4, 5), it was inferred that Ib-RCAl may not increase the activated rate of Rubisco directly, but improve the stability or activity of Ib-RCAs in photosynthesis by combination with Ib-RCAs, thereby increased activity of Rubisco indirectly and influence photosynthetic efficiency.

## Electronic supplementary material

Below is the link to the electronic supplementary material.

Supplementary material 1 (DOC 398 kb)

## Abbreviations

2-DE: Two-dimensional electrophoresis
GBSS: Granule bound starch synthase
MALDI-TOF-MS: Matrix-assisted laser desorption/ionization time of flight mass spectrometry
OEE2: Oxygen-evolving enhancer protein 2
ORF: Open reading frame
RBC: Rubisco, or ribulose-1,5-bisphosphate carboxylase/oxygenase
RBCl: Rubisco large subunit
RCA: Rubisco activase
RCAl: Long isoform RCA
RCAs: Short isoform RCA
SNP: Single nucleotide polymorphisms
