# Supplementary material for: Characterization and expression of Rubisco activase genes in Ipomoea batatas
Source: Mol Biol Rep. 2013 Sep 25;40(11):6309–21. doi: 10.1007/s11033-013-2744-7 (PMC3824211; doi:10.1007/s11033-013-2744-7)
Supplement: Supplementary file 1 — Supplementary material 1 (DOC 398 kb) [file 11033_2013_2744_MOESM1_ESM.doc]

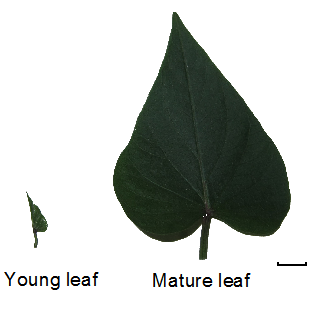


Fig. S1. A young leaf (unexpanded) and a mature leaf (fully expanded). Bar is 1 cm.

Fig. S2. The standard curves of *Ib-RCAs* (A), *Ib-RCAl* (B) and *β-actin*（C） for Real-time PCR


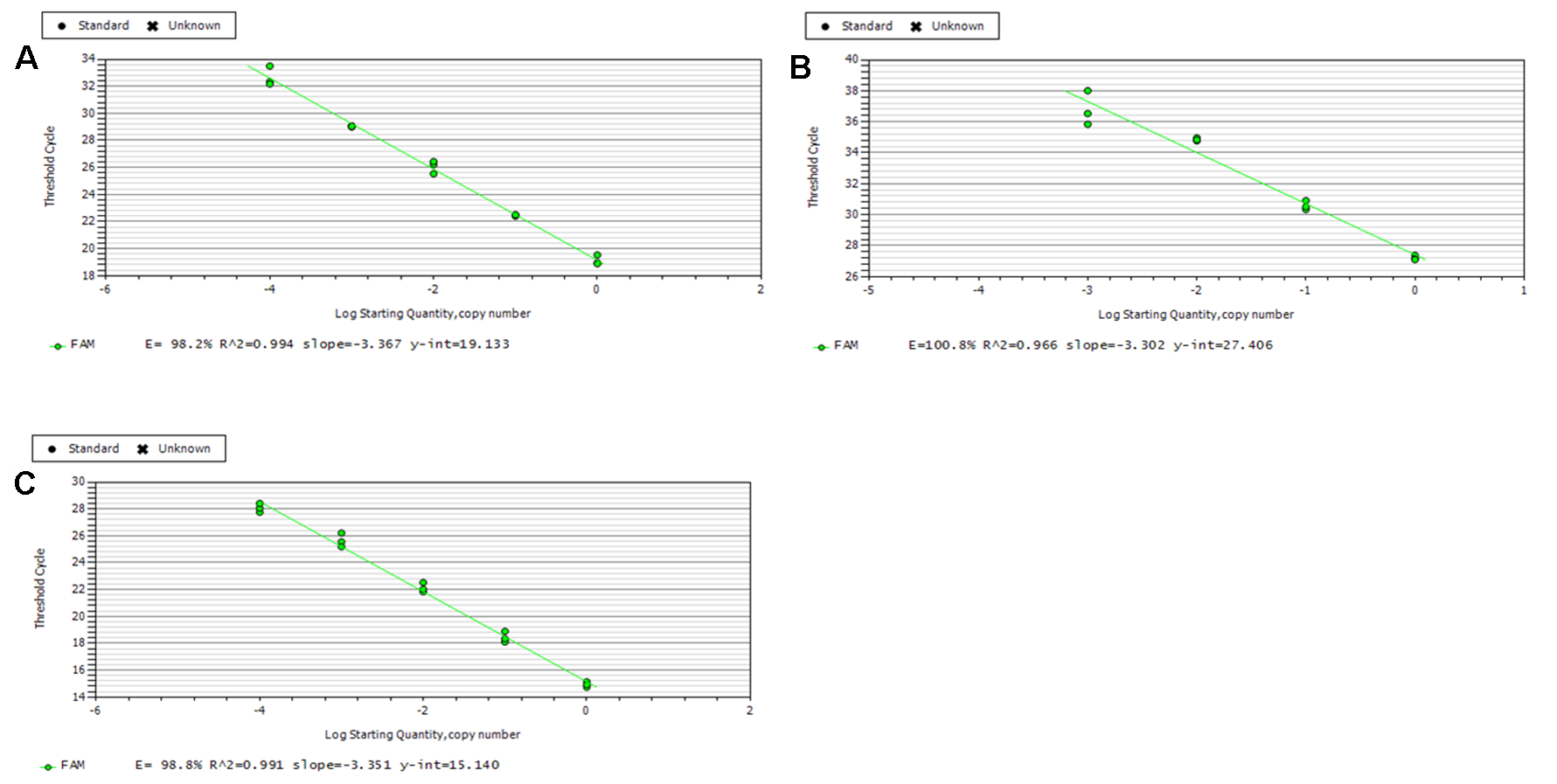


Table S1 Sequences of primers used in this work

| NO. | Primers | Sequence | PCR product length | purpose |
| --- | --- | --- | --- | --- |
| 1 | ORF-RCAsF | ATGGCTACCTCAGTGTCCACAATT | 1320 | Cloning of the ORF of *Ib-RCAs* and *Ib-RCAl* |
|  | ORF-RCAsR | TTAGCTAGCAAAAAAGGTTCCATTGTT |  |  |
| 2 | ORF-RCAlF | ATGGCTGCCACTGTCTCCAC | 1455 |  |
|  | ORF-RCAlR | TTAAACCTGGTACGTGCAGGTTC |  |  |
| 3 | Sq-5F | TCTAAGGTTTCCCCTGCCGA | 490 | *S*emiquantitative RT-PCR analysis of genes differentially expressed in young and mature leaves |
|  | Sq-5R | GCTTTCCATCGGAAACACTGG |  |  |
| 4 | Sq-7F | TTGAAGGGACAGGCTCTCCG | 299 |  |
|  | Sq-7R | TGTCTCCTCAAAGAGGATGGCA |  |  |
| 5 | Sq-11/12F | GAGCTTGTCTTCAGAAAGA TGG | 333 |  |
|  | Sq-11/12R | AGTCGTTACCGGTGACGATGA |  |  |
| 6 | Sq-13F | AAGCCTGATGGTGT CCAACG | 302 |  |
|  | Sq-13R | CAATGTCGATAGCAAAGTCACCA |  |  |
| 7 | Sq-17F | GCACA TTGTGCGGAGAGATTC | 429 |  |
|  | Sq-17R | GATGATTCCCACCAAGCCCAT |  |  |
| 8 | Sq-19F | GTGGAATGGCA GCAGGAACT | 323 |  |
|  | Sq-19R | AAACTCTCGGTGCCTCAAGTG |  |  |
| 9 | Sq-20F | TGG CTGCCAATTG AACATGAAC | 383 |  |
|  | Sq-20R | CGGTCTTCTTTCCATGGCAAAC |  |  |
| 10 | Sq-21F | GAGGAAGCC ATTCAGGCTC TC | 305 |  |
|  | Sq-21R | AGCATACTCAATTGCGGCTCC |  |  |
| 11 | Sq-25F | TCGGCGTGC TTCAGCATTTG | 252 |  |
|  | Sq-25R | TGAGGGCAAGGTGTTGAGAGTG |  |  |
| 12 | Sq-actinF | GGTGTTATGGTTGGGATGGGAC | 220 |  |
|  | Sq-actinR | GGTAAGAAGGACAGGGTGCTC |  |  |
| 13 | q- RCAsF | CTGGCAGATCAGGGCATCATCTG | 175 | Real-time PCR analyze the expression patterns of *Ib-RCAs* and *Ib-RCAl* |
|  | q- RCAsR | GGAGCTAAGAACAGCATGGTGAGTG |  |  |
| 14 | q- RCAlF | AGTACCTCAAAGAGGCTGCCCTTG | 170 |  |
|  | q- RCAlR | ACCTGGTACGTGCAGGTTCCATC |  |  |
| 15 | q-actinF | CTGGTGTTATGGTTGGGATGGGAC | 160 |  |
|  | q-actinR | GAAGGACAGGGTGCTCCTCAGG |  |  |
| 16 | BD-RBClF | CGAGAATTCATGAGTTGTAGGGAGGGATTTATGTC | 1464 | Protein-protein interaction involving Rubisco large subunit and two Ib-RCA isoforms |
|  | BD-RBClR | CGAGTCGACTTAAGCTGTACCTGGATCCAAGGT |  |  |
| 17 | BD-RCAsF | CGACCATGGGTTTCTATGGCAAGAGCTTG | 1296 |  |
|  | BD-RCAsR | CGAGGATCCTTAGCTAGCAAAAAAGGTTCCATTGTT |  |  |
| 18 | AD-RCAsF | CGACCATGGGTTTCTATGGCAAGAGCTTG | 1296 |  |
|  | AD-RCAsR | CGAGAATTCTTAGCTAGCAAAAAAGGTTCCATTGTT |  |  |
| 19 | AD-RCAlF | CGACCATGGGTTTCTTCG GGAAGGGCT | 1425 |  |
|  | AD-RCAlR | CGAGAATTCTTAAACCTGGTACGTGCAGGTTC |  |  |

Table S2 Nucleotide sequences for the partial peptide sequences used in preparing antibodies.

1. The common sequence of the two Ib-rca isoforms for the preparation of anti-RCAc antibodies

1 ATGGACAAGCTTGTTGTCCATATCACCAAGAACTTCTTGAAACTGCCCAACATTAAGATT

61 CCTCTCATCTTGGGTGTTTGGGGAGGCAAAGGTCAAGGGAAGTCTTTCCAGTGTGAGCTT

121 GTGTTCCGGAAAATGGGAATCAACCCAATTATGATGAGTGCTGGAGAACTGGAAAGTGGG

181 AATGCTGGTGAGCCAGCAAAGTTGATTAGGCAAAGGTAC

2. The sequence of *Ib-rcal* for the preparation of anti-RCAe antibodies.

1 AAGAACGGAAACTTCTATGGACAAGGAGCCCAGAGTGGTAATCTTAAGGTACCTGAAGGC

61 TGCACTGATCCTCAAGCATCAAACTTTGATCCAACCGCTAGGAGCGATGATGGAACCTGC

121 ACGTACCAGGTTTAA

Table S3 Categories of differentially expressed proteins in young and mature leaves.

| categories | proteins |
| --- | --- |
| Photosynthesis | carbonic anhydrase; contains β-transducin motif; F-type H^+^-transporting ATPase subunit γ; granule bound starch synthase; oxygen evolving enhancer protein 2; phosphoribulokinase; phytochrome B 2; ribulose-bisphosphate carboxylase large chain; rubisco activase |
| Energy metabolism | acetyl-CoA carboxylase/biotin carboxylase; fructose-bisphosphate aldolase; nucleoside diphosphate kinase; phosphoglycerate kinase precursor |
| Transcription regulation | elongation factor 1-α; putative glycine-rich RNA-binding protein 2 |
| Stress-responsive | cyclophilin; stromal 70 kDa heat shock-related protein |
| Skeleton protein | actin 3 |
| Transport protein | zinc/iron transporter |

Table S4 Difference with three similar isoforms of *Ib-RCAs* and *Ib-RCAl*

| Mutational site | 81 | 121 | 172 | 591 | 600 | 735 | 870 | 888 | 933 | 936 | 965 | 1221 |
| --- | --- | --- | --- | --- | --- | --- | --- | --- | --- | --- | --- | --- |
| *Ib-RCAs I* | T | T | C | C | G | T | A | C | C | A | G | C |
| *Ib-RCAs II* | C | C | A | T | C | C | T | T | T | C | G | T |
| *Ib-RCAs III* | C | C | C | T | G | T | T | T | T | A | A | C |
| Codon^a^ | 3 | 3 | 1 | 3 | 3 | 3 | 3 | 3 | 3 | 3 | 2 | 3 |
| Amino acid^b^ | N | P to S | Q to K | N | N | N | N | N | N | N | R to K | N |

| Mutational site | 121 | 142 | 249 | 310 | 336 | 363 | 372 | 378 | 390 | 411 | 435 | 624 | 627 |
| --- | --- | --- | --- | --- | --- | --- | --- | --- | --- | --- | --- | --- | --- |
| *Ib-RCAl I* | A | T | G | T | T | A | C | C | C | A | A | T | T |
| *Ib-RCAl II* | A | C | G | T | C | T | T | T | T | G | T | C | C |
| *Ib-RCAl III* | G | C | A | A | C | T | T | T | T | G | T | C | C |
| Codon^a^ | 3 | 3 | 3 | 1 | 3 | 3 | 3 | 3 | 3 | 3 | 3 | 3 | 3 |
| Amino acid^b^ | N | P to S | N | S to T | N | N | N | N | N | N | N | N | N |

| Mutational site | 714 | 720 | 734 | 747 | 792 | 939 | 951 | 1044 | 1098 | 1140 | 1164 | 1302 | 1326 |
| --- | --- | --- | --- | --- | --- | --- | --- | --- | --- | --- | --- | --- | --- |
| *Ib-RCAl I* | T | T | G | G | A | C | A | A | G | T | C | T | T |
| *Ib-RCAl II* | T | C | A | A | T | T | T | C | A | C | T | C | C |
| *Ib-RCAl III* | T | C | G | A | T | T | T | C | A | C | T | C | C |
| Codon^a^ | 3 | 3 | 1 | 3 | 3 | 3 | 3 | 3 | 3 | 3 | 3 | 3 | 3 |
| Amino acid^b^ | N | N | G to E | N | N | N | N | N | N | N | N | N | N |

^a^ Position in a codon.

^b^ N, No change in amino acid. P to S, proline to serine change
